# Supplementary material for: Afferent specific role of NMDA receptors for the circuit integration of hippocampal neurogliaform cells
Source: Nat Commun. 2017 Jul 28;8:152. doi: 10.1038/s41467-017-00218-y (PMC5532276; doi:10.1038/s41467-017-00218-y)
Supplement: Supplementary file 1 — Supplementary Information [file 41467_2017_218_MOESM1_ESM.pdf]

File Name: Supplementary Information
Description: Supplementary Figures and Supplementary Table.

File Name: Peer Review File
Description:

**Supplementary Figure 1**

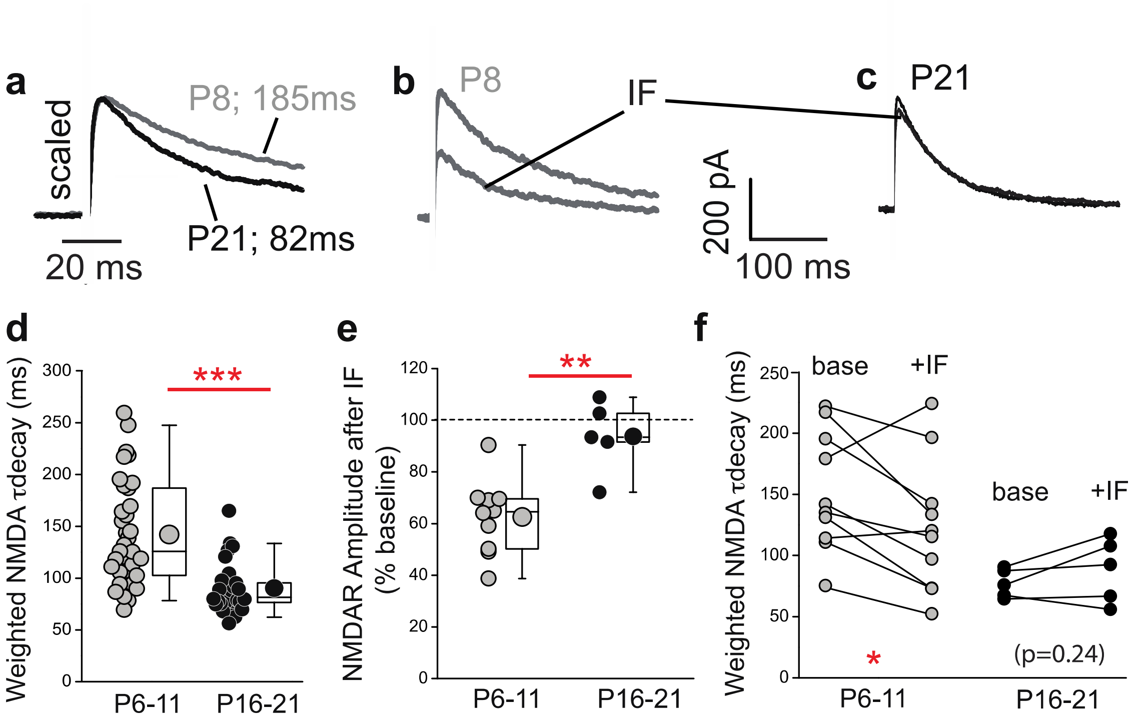


**NMDAR-mediated EPSCs elicited by SLM stimulation onto CGE-derived NGFCs undergo an NR2B to NR2A switch during postnatal development. (a-c)** Single trace examples of NMDAR-mediated EPSCs illustrating decay kinetics and sensitivity to ifenprodil (IF, 5μM) at P6-11 (gray traces) and P16-21 (black traces). **(d)** Weighted τ decay values of NMDAR EPSCs at P6-11 (gray circles; n=36) versus P16-21 (black circles; n=31). **(e)** IF sensitivity of NMDAR-mediated EPSC at P6-11 (gray circles; n=10) versus P16-21 (black circles; n=5). **(f)** Effects of IF on the decay kinetics of NMDAR-mediated EPSC at P6-11 (gray circles; n=10) and P16-21 (black circles; n=5). Connecting lines denote weighted τ decay values measured before and after IF in individual NGFCs. Mann Whitney U-tests were used for all comparisons except in (f) where a Wilcoxon test was used (**p<0.01, ***p<0.001). All n values correspond to the number of cells recorded. Construction of box-whisker plots is detailed in methods.

## Supplementary Figure 2

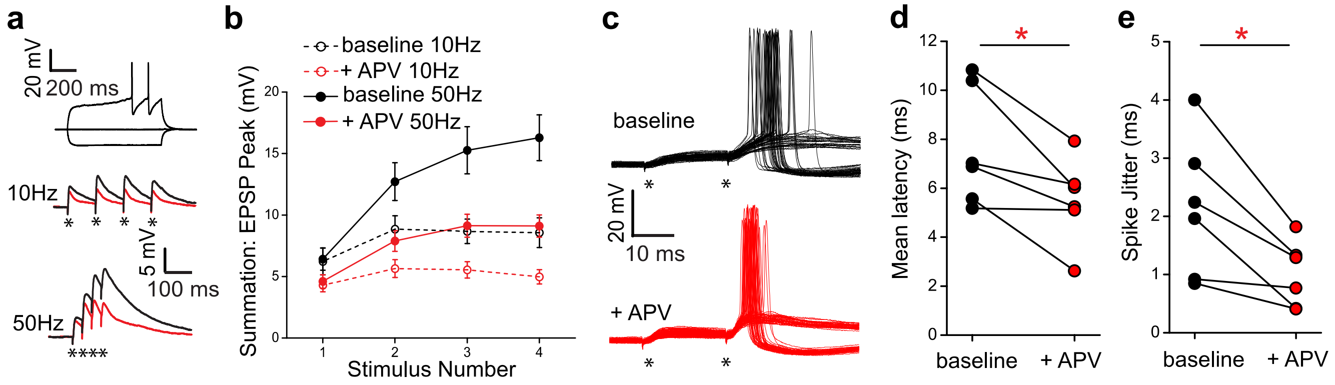


**SLM afferent evoked NMDAR-signaling onto NGFCs is critical for their recruitment and spike timing. (a)** Single trace example of voltage responses to current steps in SLM NGFCs recorded in the *Htr3A*-cre:Ai14 mouse line demonstrating lack of sag at hyperpolarizing step and late-spiking phenotype at threshold current injection (top trace). Single traces illustrate EPSP summation to SLM stimulation (4 stimulations at 10 and 50 Hz; middle and bottom traces, respectively; asterisks denote time of each stimulation) under control conditions (black) and in the presence of APV (red). **(b)** Pooled data of EPSP summation during trains of 4 stimuli at 10 and 50 Hz in control conditions and in presence of APV (n =5). **(c)** Overlaid traces (50 consecutive sweeps) illustrating SLM afferent evoked EPSP-spike coupling (2 stimulations at 50 Hz; asterisks denote time of stimulation) in an NGFC under control conditions (baseline; top black trace; stimulation intensity set to produce approximately 50% spike success after second stimulation) and in the presence of APV (bottom red trace). Note: after APV application stimulation intensity was re-adjusted to maintain 50% spike success rate **(d,e)** Line plots indicating the effect of APV on the mean latency and spike jitter in individual NGFCs (n=5). Wilcoxon test was employed (*p<0.05) and all n values correspond to the number of cells recorded. Error bars denote SEM.

**Supplementary Figure 3**

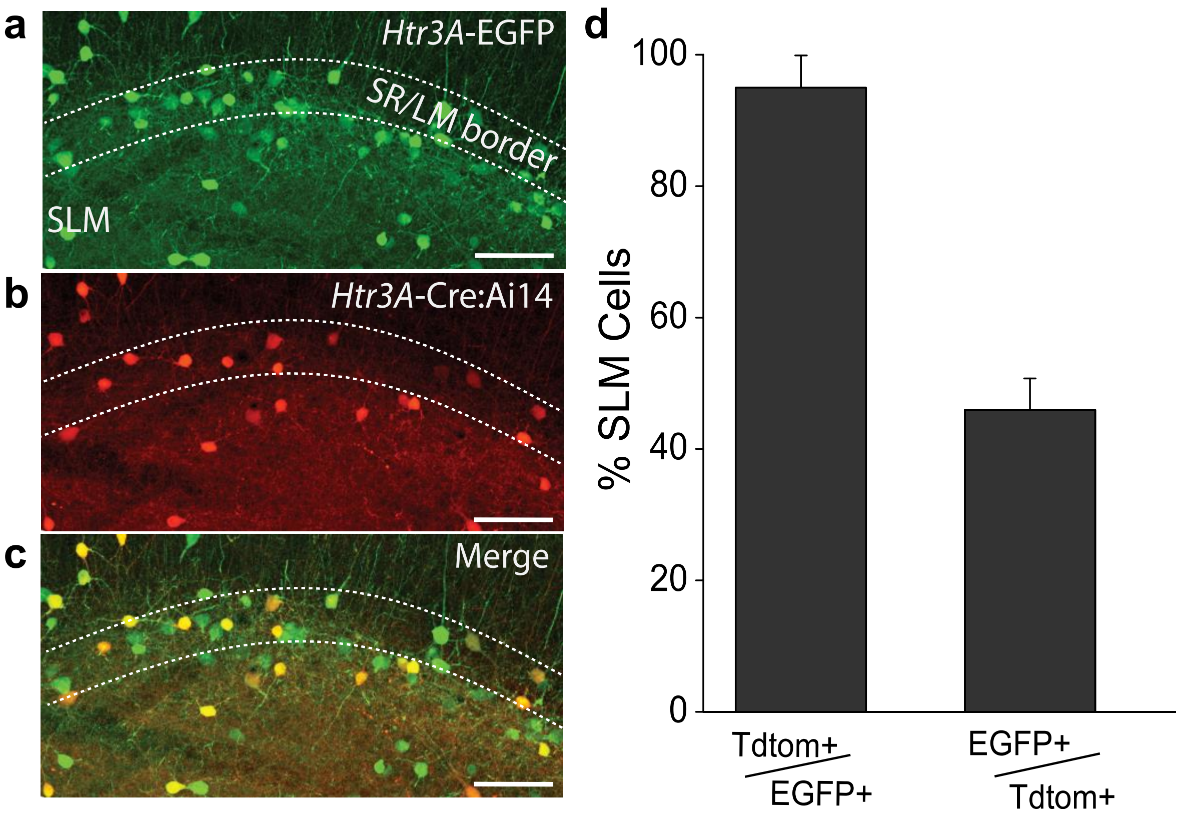


**Comparison of extent to which SLM interneuron cells are reported in the *Htr3A*-EGFP versus the *Htr3A*-cre:Ai14 transgenic mouse lines. (a)** Confocal image of hippocampal SLM region in a crossed Htr3A-EGFP and Htr3A-Cre:Ai14 mouse line. (b). Cell counts in SLM illustrating virtually all Cre-expressing TdTom cells are EGFP-positive whereas only approximately half of EGFP-positive INs express TdTom (number of mice = 3; 8-10 hippocampal sections were counted per mouse; number of cells counted per hippocampus = 7 – 21; the whole SLM region was analyzed). Error bars denote SEM.

**Supplementary Figure 4**

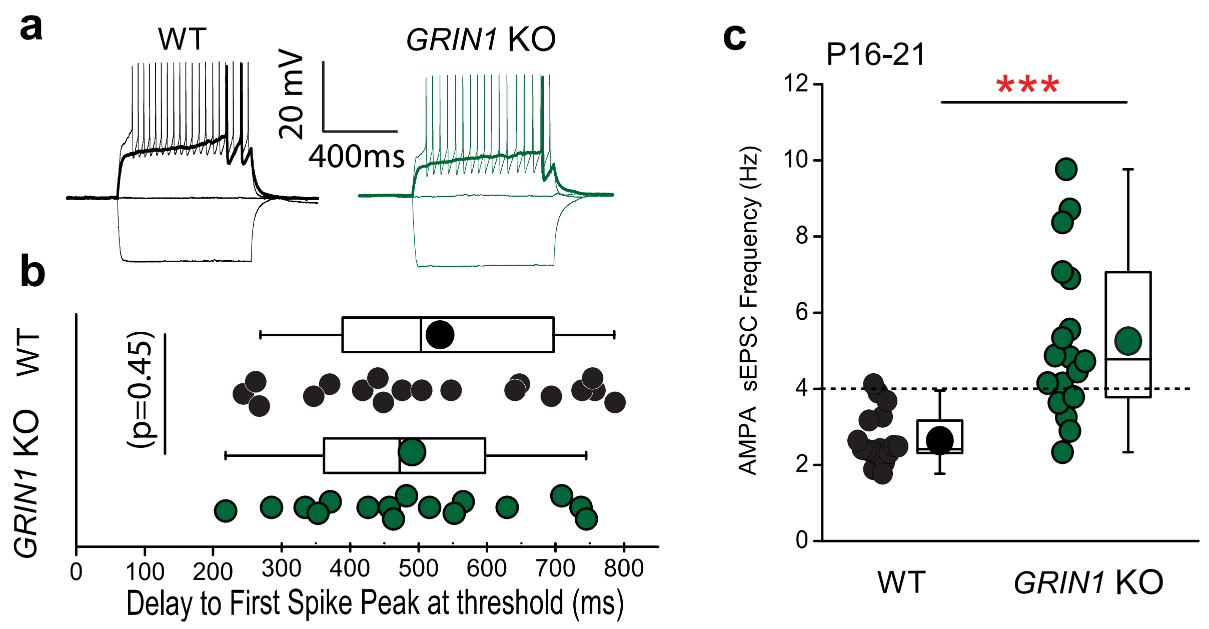


**Identification of late-spiking CGE-derived NGFCs in SLM of WT and *Grin1* KO mice. (a)** Single trace examples of voltage responses to current steps in CGE NGFCs of SLM from P16-21 WT (black traces) and *Grin1* KO mice (green traces) demonstrating lack of sag at hyperpolarizing step, late-spiking phenotype at threshold current step (bold traces) and action potential output at 2 X threshold step. **(b)** First spike latency at threshold current injection in NGFCs subsequently analyzed for $sEPSC_{AMPA}$ frequency (n = 18, 16 for WT and *Grin1* KO, respectively). **(c)** $sEPSC_{AMPA}$ frequency of late-spiking NGFCs in P16-21 WT and *Grin1 KO* mice (n =18,16). Mann Whitney U-tests were used for comparison (***p<0.001). All n values correspond to the number of cells recorded. Construction of box-whisker plots is detailed in methods.

## Supplementary Figure 5

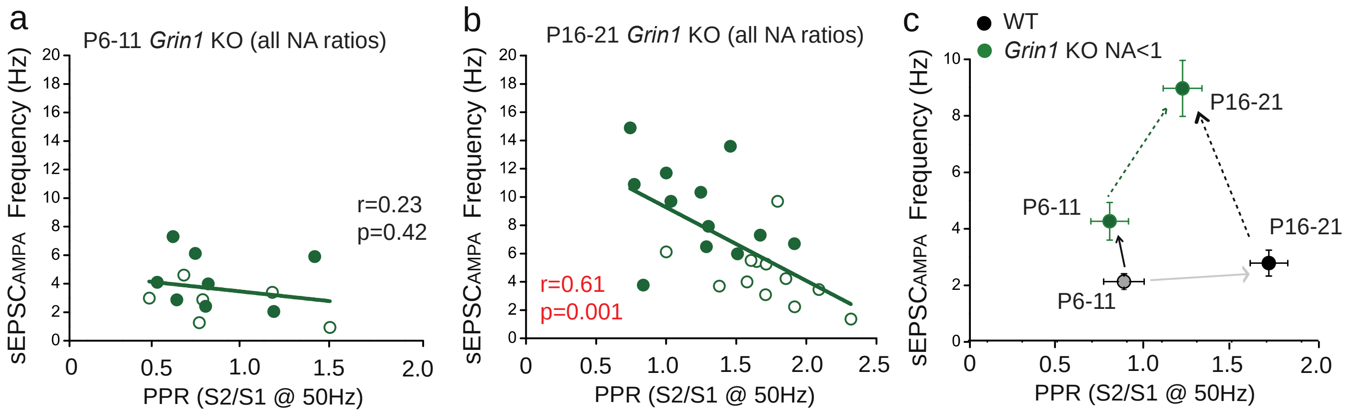


**Correlation analyses of PPR and sEPSC$_{AMPA}$ frequency measured in individual NGFCs in *Grin1* KO mice during postnatal development.** (**a,b**) Correlation of PPR and sEPSC$_{AMPA}$ frequency in NGFCs (all NA ratios; NA<1, filled green circles; NA>1, open green circles) from P6-11 and P16-21 *Grin*1 KO mice. r and p values as indicated from straight-line fit analyses demonstrate a significant positive linear correlation for the P16-21 developmental age. **(c)** Mean values of PPR and sEPSC$_{AMPA}$ frequency in NGFCs from WT (black) and *Grin1* KO (green; NA<1) as the ages indicated. x and y error bars denote SEM. Please refer to Results section for full description of arrows depicted in this panel. All n values correspond to the number of cells recorded.

## Supplementary Figure 6

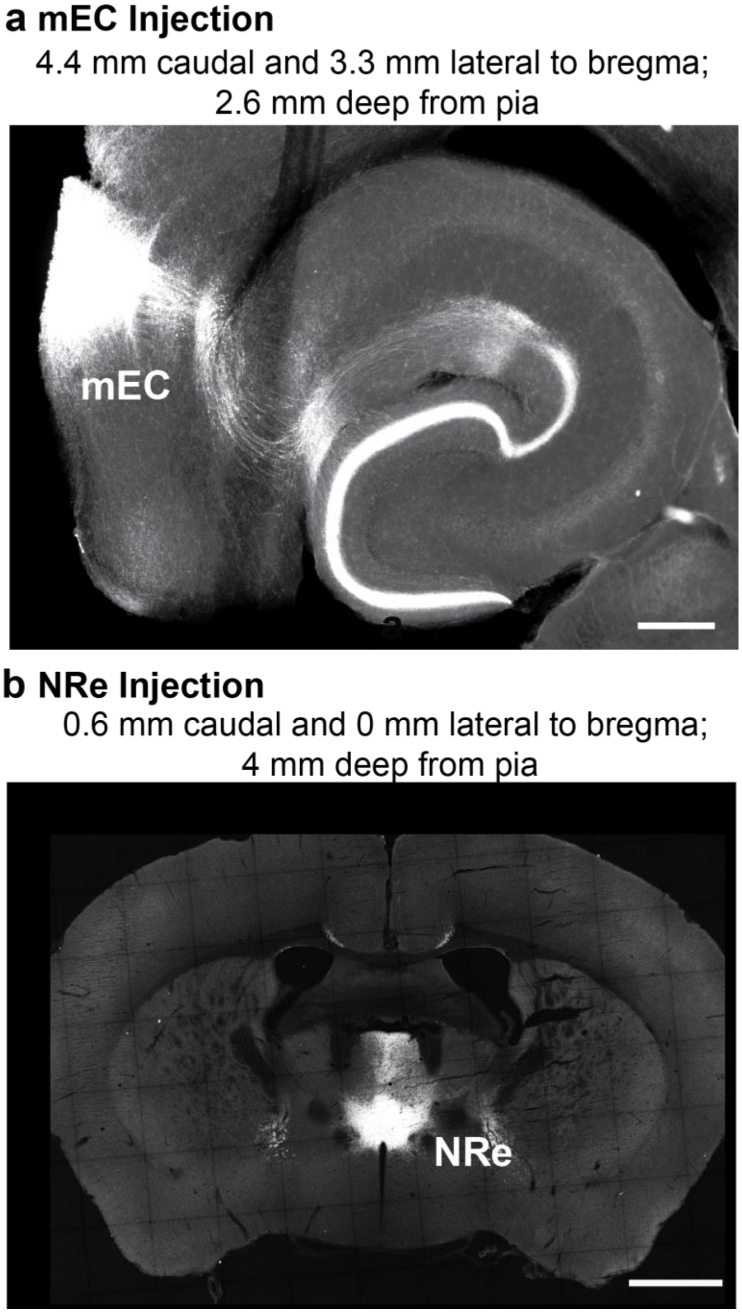


***In vivo* viral injections of channelrhodopsin into regions of mEC and NRe employed to optogenetically dissect extrahippocampal inputs onto NGFCs. (a,b)** Confocal images of brain sections illustrating channel rhodopsin expression after *in vivo* viral injections (2 weeks post injection) to target regions of mEC and NRe employing the coordinates indicated. Please refer to methods for detailed description of the injection protocol. Scale bars in **(a)** and **(b)** denote 100 μm and 1 mm, respectively.

## Supplementary Figure 7

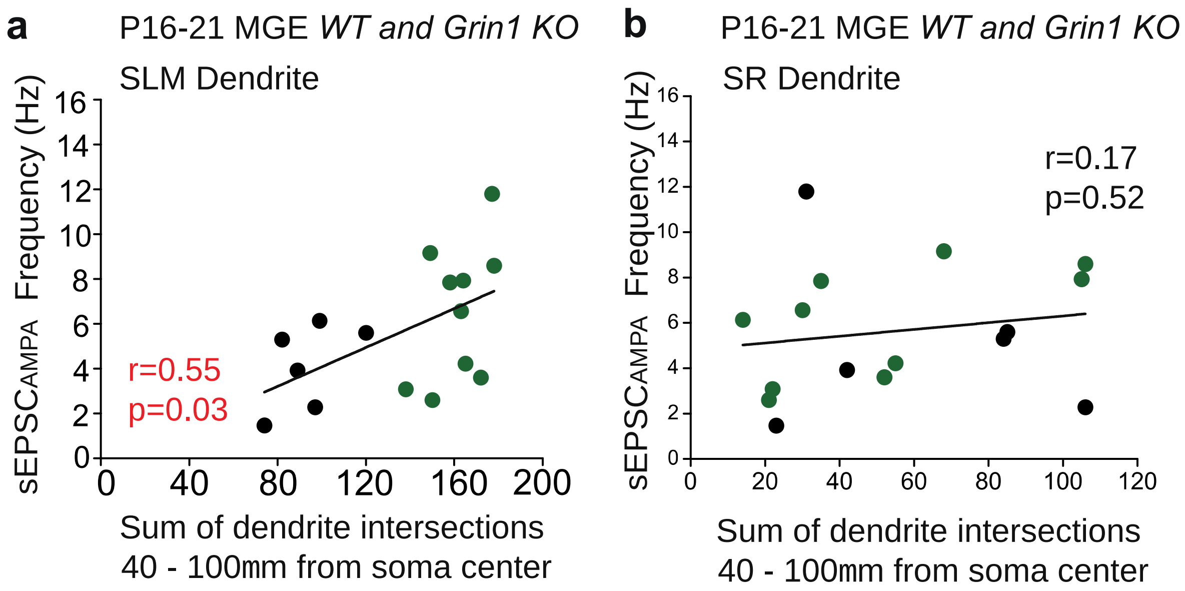


**Correlation analyses of dendrite complexity and sEPSC$_{AMPA}$ frequency measured in individual MGE-derived NGFCs in *Grin1* KO mice during postnatal development. (a,b)** Correlation between the sum of dendrite intersections (40 – 100 µm from the soma center) and sEPSC$_{AMPA}$ frequency in individual NGFCs for the SLM and SR input. Black and green symbols denote observations from MGE WT and MGE Grin1 KO NGFCs. r and p values as indicated from straight-line fit analyses demonstrate a significant positive linear correlation for SLM inputs only.

**Supplementary Table 1**

| | *Htr3ACre:*Ai14 WT (n-16) | *Htr3ACre:*Ai14 *Grin1* KO (n-13) | *Mann-Whitney* U-test p-value |
|---|---|---|---|
| **First Spike Latency @ threshold (ms)** | 490 ± 39 | 549 ± 45 | 0.25 |
| **sEPSC AMPA Frequency (Hz)** | 2.6 ± 0.2 | 5.9 ± 0.5 | <0.001 |
| **Input Resistance (mΩ)** | 231 ± 21 | 269 ± 22 | 0.18 |
| **Rheobase (pA)** | 106 ± 12 | 83 ± 11 | 0.08 |
| **Membrane Time Constant (ms)** | 22 ± 1.4 | 18 ± 2.0 | 0.31 |
| **Capacitance (pF)** | 101 ± 6.8 | 98 ± 6.0 | 0.94 |
| **Firing Frequency @ 2 X threshold (Hz)** | 20 ± 1.4 | 18 ± 1.9 | 0.28 |
| **Resting Membrane Potential (mV)** | -73.4 ± 1.2 | -75.4 ± 1.3 | 0.24 |
| **Sag Index** | 0.99 ± 0.002 | 0.99 ± 0.004 | 0.12 |

**Summary of firing and membrane properties of late-spiking CGE-derived NGFCs in SLM of WT and *Grin1 KO* mice.**
